# Supplementary material for: Baculovirus-Free SARS-CoV-2 Virus-like Particle Production in Insect Cells for Rapid Neutralization Assessment
Source: Viruses. 2022 Sep 20;14(10):2087. doi: 10.3390/v14102087 (PMC9606917; doi:10.3390/v14102087)
Supplement: Supplementary file 1 [file viruses-14-02087-s001.zip › viruses-1914242-supplementary.pdf]

Supplement

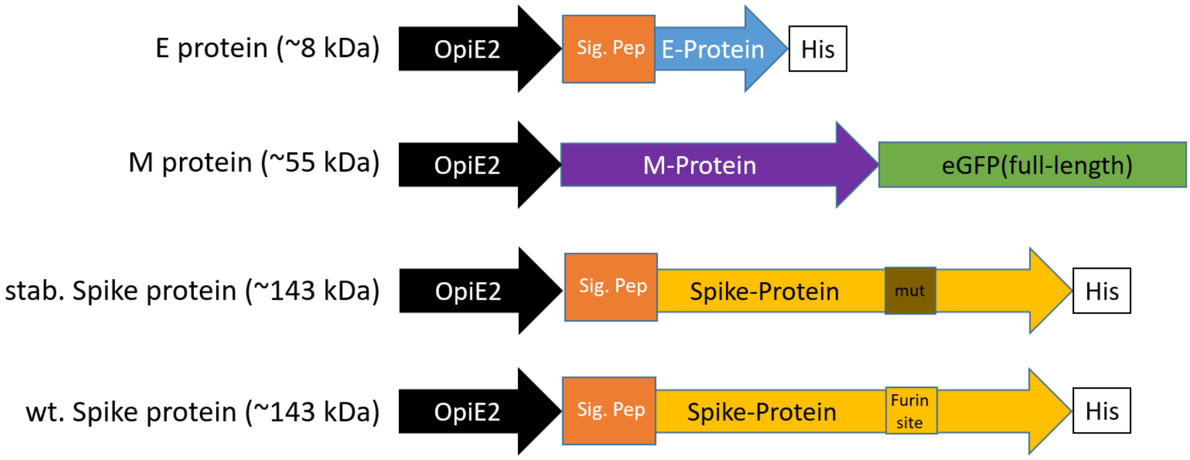

Supplementary Figure S1. Overview about the expression constructs used in this study after optimization. In all VLP variants the stabilized Spike version (stabilized by proline substitutions at position 986 and 987 and "GSAS" substitution at the Furin site (residues 682–685 aa)) was used with exception of VLP-6M-Furin, where the wildtype version was employed.

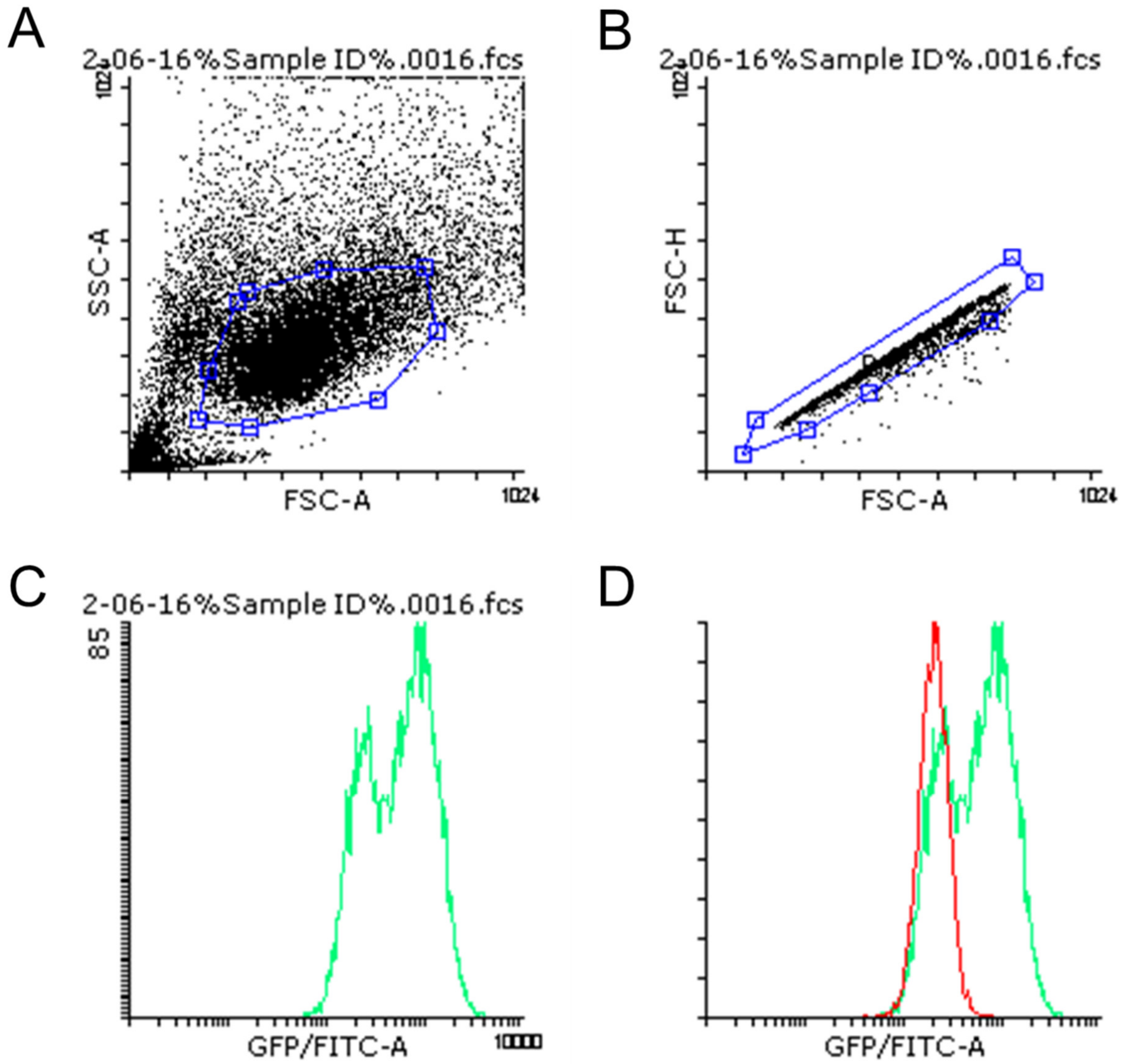

Supplementary Figure S2. Gating strategy in cytometry assay. A) Gate shown to select living cells, B) gate selects out of the living cell population the single cells by FSC Height vs. Area C) shows the resulting GFP signal of single cells (10000 events measured) D) Histogram Overlay of the GFP signal of single cells of VLPs binding to ACE2 negative cells (red) or ACE2 positive cells (green) where two distinct populations are visible. Analysis shown here was performed using Flowing Software 2, analysis in FlowJo was identical.

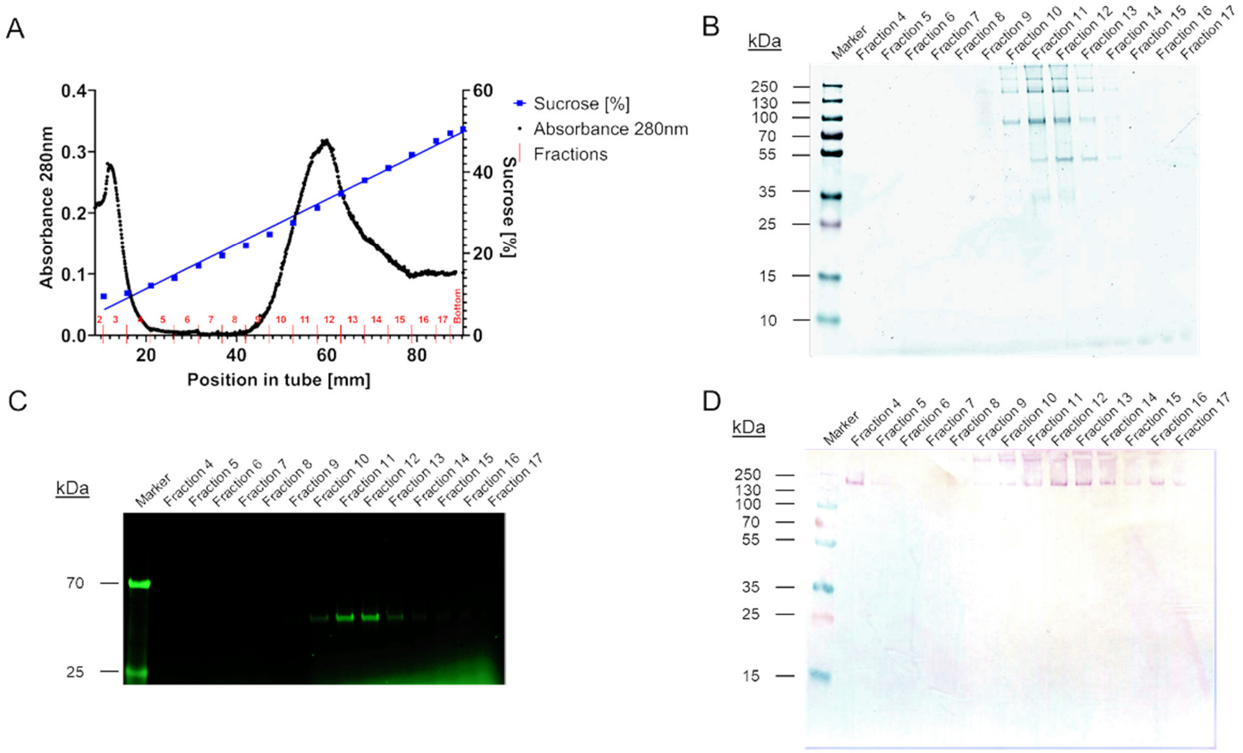

Supplementary Figure S3. VLP-6M purified by 10-50% sucrose gradient centrifugation (A) were analysed by SDS PAGE (B). C) In-gel GFP fluorescence analysis to visualize M protein-GFP fusion protein that is expected to run at ~55 kDa and D) anti-His Immunoblot of fraction 1-12. Monomeric spike protein at ~143 kDa and trimeric spike at ~429 kDa (mass without glycosylation) can be detected, whereas E protein at ~8 kDa is not observed.

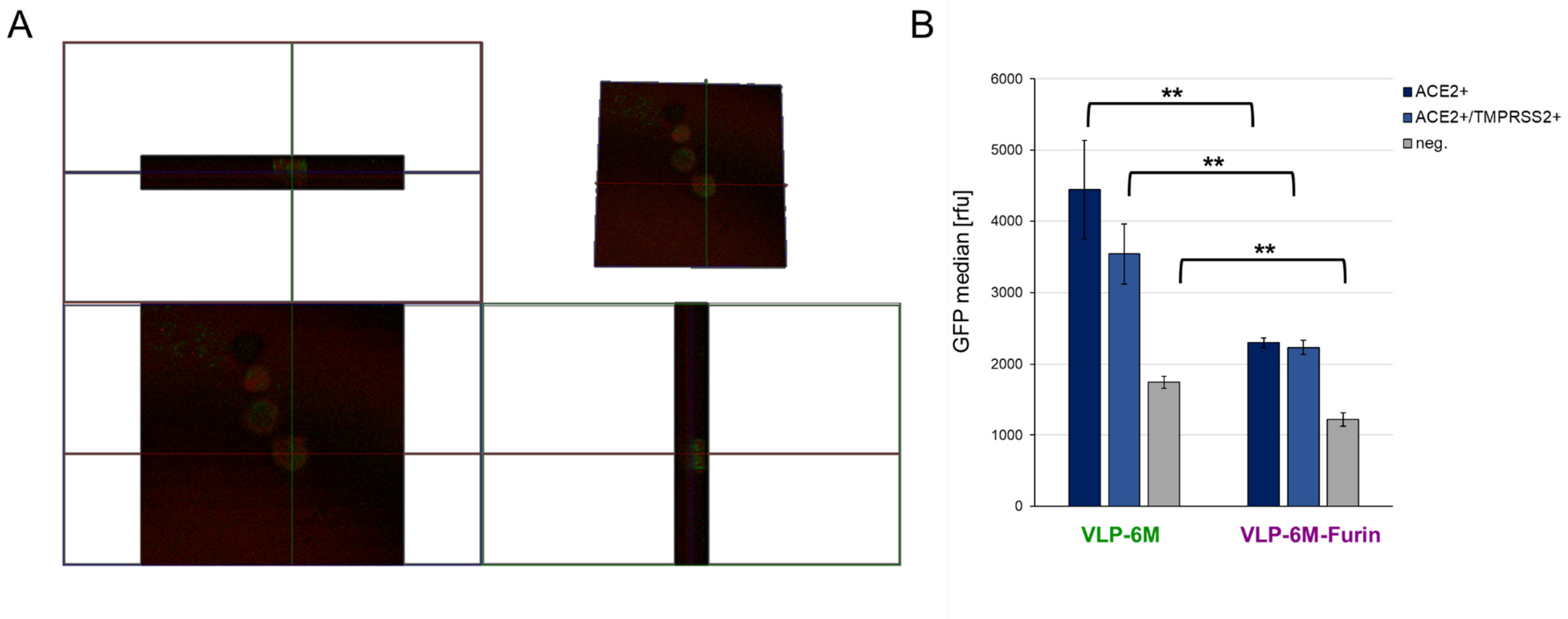

Supplementary Figure S4. A) 3D image of a single ACE2 and TMPRSS2 positive cells that seemed to have taken up VLP-6M-Furin. Such uptake could only be observed for some individual cells. B) GFP median signal to noise ratios of the VLP binding ACE2 or ACE2 and TMPRSS2 expressing Expi293F cells versus ACE2 negative cells determined by cytofluorometry. Error bars indicate the standard deviation between three independently transfected Expi293F cell populations. Significances were determined by two sided T-Test (* = 90%, ** = 95%).

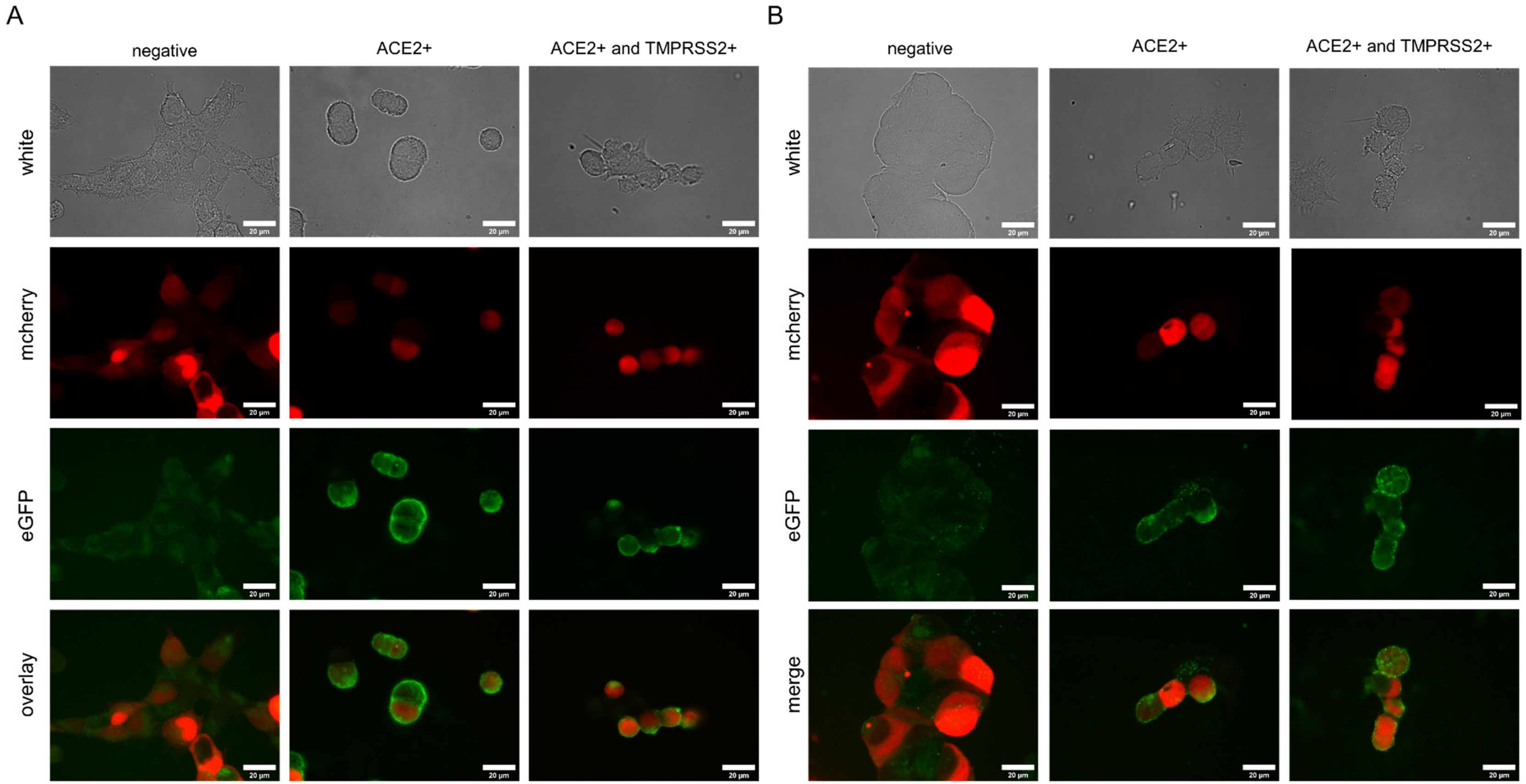

Supplementary Figure S5. Bright field, GFP, mCherry and merged microscopy images of VLP-6M (A) and VLP-6M-Furin (B) binding to ACE2 negative, positive or ACE2 and TMPRSS2 positive Expi293F cells.

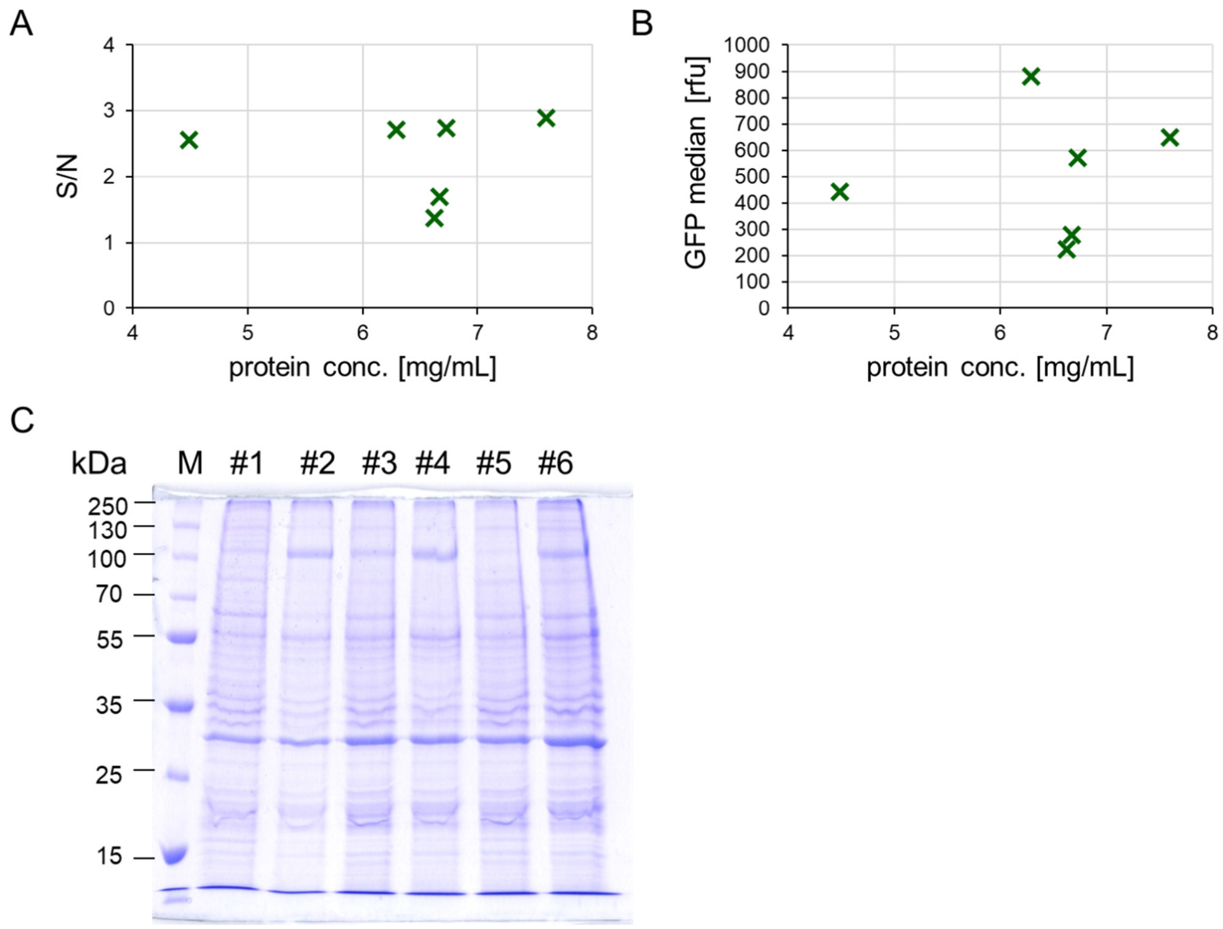

Supplementary Figure S6. Comparison of different VLP-6M batches. Correlation of the measured protein concentration (Abs. 280 nm) of the concentrated VLP of different VLP batches and A) signal to noise ratio in cytometry assay on ACE2 expressing cells versus ACE2 negative cells or B) GFP median signal of single ACE2 positive cells. C) SDS PAGE (12%) of the individual VLP batches. 10 µL of concentrated VLP were applied. Monomeric spike protein has a size of ~143 kDa and trimeric spike at ~429 kDa (mass without glycosylation) E protein at ~8 kDa.

Supplementary Table S1. Details of the used serum samples.

	**Individuals (f/m)**	**Days after Vaccination**
PreCorona	1 (1/0)	-
1x Ad26.COV2.S	4 (1/3)	14-33 (mean 24)
2x BNT162b2	4 (1/3)	12-13 (mean 12.5)
3x BNT162b2	4 (4/0)	12-13 (mean 12.75)
